# Meteorological Table

**Published:** 1859-03

**Authors:** J. G. Westmoreland

**Affiliations:** Smithsonian Institution, February, 1859, at Atlanta,Ga.


					﻿•*-> 1
-
S	. is £ S ■ . . b 3:	. . -3 .	3 ft ft
QO	rn . . . jJZJKM .	. £ £ . ...fetn«tri.
T—O	CQ Zi Zi Zi	2; z	02 CO W2	027202
£
<3	--------- -------------------------------------------------
SJ
~O
Stu	!	®	*	>' > >	>. •	■>.' J. ■'	u-	•	•
,.Jo I £ i «2x . ..ZaSZ, . gX	,jS	,h". ZcXXCQ"^
St	O1	Z Z Zi	Z 02 Z 02 X 02 02 Z	02 02
•« <2
a ‘O
.-= O
■ii T—(	----------
co	I	I
jS rT
V	S	‘	> 5J	■ . •	- ■............■	•	•
|	1-	Z	02 z Zxtnx’xxxxZ	02 x
1- '
cs §------------- ------------------------------------------------—
g ~
d$	s
<r> ’’S	•	“°® 0 ° — 0 — 2 10 00 ° 10 10 0 0 °‘®	© © © OOC5 O _> O
.©>	r-H	—> -H r-(	— rH rH r-< r-< i
\i L -
ro	a	•
0 TO	P	a
’h o	O	•	10 2 O ~	'&	©' © © O >© KO O >0000 >0 >0 >000000000
. t — I i ft_q	P«	'	—< r—! T—<	—I r-l r-> ftH r-f —,
I 0	M
^3 I
"S.-5	"	----------'I
ftS &	»
V O	•	'-"^ 2	0 0 0 ~ °	— 0 O O C3 >O O O O O uoo UO o o o 00 o
_ __ ,_, ^,r_r-|r_
SI
CO *X —
3	°	£	£ £S	.£ S
q$*______________*____2	©	©	©~	00
.J> I .	»	----------I
2h	-•	2 Ti J rc 2 S 2. J ~	O © Cl O Cl H’ CC' H”f Cl O Cl — Cl H1
52	—	^’tz3c003^^,coe'302®3'^l'+l'^<i0>0l0i000-fl-t<>0>0 00l0l0l0 I
J^’-S	El ©	_
•° c	H	“K------- . ------------------------------------------------
o «•	§ 3 co $5 2 £< 2 2 2 2 2 22 0	0 00 01 00	0 °	0 '
1	“ °	’t iO	Tf^ 1O 1O 1^ N 1.0 ^ O tc O O lO 1Q O
■S d I a -^L -_________ _ I
co	a	s
.© (< S	j	00030000:>’OO'*CX
-S	“	V JWrorown’M't*'#50iOfflu5cOM'^iOiOCO>0'0‘a|
« S I____________
t -2	SS .	10®0>OC»COOOOJaO	H-	>n m ft_
.	oj 01 oj © .co co ojcocoojcot^TjjcQ^Hftjfcq 01 >o> w coco mh
-O '	rv“. ®*	S SSSSSSSS00©©©© o' o' OOOOOO OOOOO 1
Qj	1 g.	CO co <N ©• 05 ^101 02 01 01 CO co co co oi CO CO CO CO CO CO CO 5ci O1 02 <N co I
•*»	63 S . 00«© „,2.co 00 O Tfl	Tao	i>- t- m
Q	Ch	10 O1 02 CO .1 CO cool CO CO Mi	t-H 1O CO 0<l CO CO co COCOCOCO'-1
*°**	1	_	5 O’ O O o O <0 O >*~*^ C7s c*"*^	z**5 d d si
O	W^^^^’^^^^^WCOCOCOCOCOCOCQWClWCOCOGqC^^OlCO 1
pc? _E?_ ------------- --------------------------------------------(
O	.	« ^^WOXCOQ^XO	00 00	t—	00 N 511 O N
PQ	<:<?c^::^^c^c^c^c^C<!^,^J'^^iOCOCO^ClC<lCOd>^C<'ICOCQeOCQCQ
!	•<	$	O O O O O q O O O Ci O O O Oi oi O Q iTi
**<•	11	.	^^^^^^^^^^IWCOCOCOiMlMCOCOeO^eOCOCQCICKNC^N
<0	I________I”
33 I Dav of Mo. '“<o*c,9-v<iocol— cocse>I-<cqco'tf<»G>cc>j>.aoc» Si rH 6o Mtio* co t- ad
’	11 J	T-H f—< 1—I rH rH M H rH M Cl Cl Cl Cl O1 Cl 02 O1 O I
				

